# Anthropometric Measurements Inform Complete Concentric Collapse Status in Patients With Obstructive Sleep Apnea

**DOI:** 10.1002/oto2.70245

**Published:** 2026-05-05

**Authors:** Jordan S. Weiner, Eugene G. Chio, Raj C. Dedhia, Colin T. Huntley, Michael J. Hutz, Phillip Huyett, Daniel J. Lee, Philip LoSavio, Ilya Perepelitsyn, Ryan J. Soose, David T. Kent

**Affiliations:** ^1^ Valley ENT Scottsdale Arizona USA; ^2^ Department of Otolaryngology The Ohio State University Wexner Medical Center Columbus Ohio USA; ^3^ Division of Sleep Surgery, Department of Otorhinolaryngology–Head & Neck Surgery University of Pennsylvania Philadelphia Pennsylvania USA; ^4^ Divsion of Sleep Medicine and Surgery, Department of Otolaryngology–Head & Neck Surgery Thomas Jefferson University Philadelphia Pennsylvania USA; ^5^ Division of Sleep Surgery, Department of Otorhinolaryngology Rush University Chicago Illinois USA; ^6^ Division of Sleep Medicine and Surgery, Department of Otolaryngology–Head & Neck Surgery Massachusetts Eye and Ear Boston Massachusetts USA; ^7^ Division of Otolaryngology–Head & Neck Surgery, Department of ENT and Allergy Mason City Clinic Mason City Iowa USA; ^8^ Division of Sleep Surgery/General Otolaryngology, Department of Otolaryngology ENT Specialty Care Minneapolis Minnesota USA; ^9^ Division of Sleep Medicine and Upper Airway Surgery, Department of Otolaryngology University of Pittsburgh Pittsburgh Pennsylvania USA; ^10^ Division of Sleep Surgery, Department of Otolaryngology–Head & Neck Surgery Vanderbilt University Nashville Tennessee USA

**Keywords:** complete concentric palatal collapse, drug induced sleep endoscopy, hypoglossal nerve stimulation, lateral pharyngeal wall collapse, obstructive sleep apnea, supine pharyngeal width

## Abstract

**Objective:**

Hypoglossal nerve stimulation in the US requires drug‐induced sleep endoscopy to exclude patients with complete concentric collapse. This is an expensive and time‐consuming requirement. We hypothesized that supine pharyngeal width, and other demographic and polysomnographic variables would associate with complete concentric collapse.

**Study Design:**

Prospective, multicenter cohort study.

**Setting:**

10 centers in the United States with experience selecting patients for and performing airway surgeries for sleep apnea including hypoglossal nerve stimulation implantation.

**Methods:**

600 patients meeting criteria for hypoglossal nerve stimulation underwent measurement of supine pharyngeal width and collection of demographic and polysomnographic data followed by drug‐induced sleep endoscopy.

**Results:**

587 patients completed the study. Patients with complete concentric collapse had a higher body mass index (31.2 ± 3.2 vs 29.0 ± 3.4 kg/m^2^, *P* < .001), larger neck circumference (45.5 ± 4.2 vs 40.6 ± 4.7 cm, *P* < .001), and lower supine pharyngeal width (19.4 ± 6.3 vs 21.8 ± 6.5 mm; *P* = .008) than patients without complete concentric collapse.

**Conclusion:**

Body mass index, neck circumference, and supine pharyngeal width all associate with complete concentric collapse and could potentially be used to determine hypoglossal nerve stimulation candidacy instead of drug‐induced sleep endoscopy for most patients thereby reducing both time and cost. (ClinicalTrials.gov NCT05428839: https://clinicaltrials.gov/study/NCT05428839?term=Inspire%20Medical%20systems%20predictor&rank=1).

Obstructive sleep apnea (OSA) is a prevalent condition associated with an increased risk of motor vehicle and workplace accidents^1^, ^2^ as well as cardiovascular morbidity and mortality.^3^ Treatment is recommended for both reduction of symptoms and to reduce long‐term morbidity and mortality from associated comorbid conditions.^4^, ^5^, ^6^ Continuous positive airway pressure (CPAP) is the standard first‐line treatment^5^, ^7^ but many patients are unwilling or unable to comply with therapy.^8^ Hypoglossal nerve stimulation (HNS) is an alternative surgical therapy for select CPAP‐intolerant patients with moderate to severe OSA.^9^ HNS protrudes the tongue to reduce upper airway collapsibility, which dilates the pharynx at multiple levels through elastic mechanical linkages to other airway structures, including the soft palate.^10^

Prior research indicates that HNS nonresponders experience persistent airflow limitation despite maximal tolerable tongue protrusion^10^, ^11^, ^12^, ^13^, ^14^, ^15^, ^16^ due to persistent collapse of the soft palate or lateral oropharyngeal walls.^16^ HNS candidacy criteria currently require drug‐induced sleep endoscopy (DISE) to rule out complete concentric palatal collapse (CCC), as it was found to be predictive of HNS non‐response during an early feasibility trial.^9^, ^16^ Although studies are limited due to existing FDA indications excluding patients CCC, data has been published demonstrating improvement in CCC patients.^17^ Nevertheless, DISE is time‐consuming, expensive, requires intravenous anesthesia, and only has moderate interrater reliability for assessment of the degree and pattern of palatal and lateral wall collapse.^18^, ^19^, ^20^, ^21^

The transverse width of the pharynx affects its collapsibility. A narrow, high‐arched palate is more common in patients with OSA^22^ and is associated with the presence of CCC, tongue base collapse, and increased pharyngeal closing and opening pressures.^23^, ^24^ Surgery to widen the maxilla increases nasal width and pharyngeal volume, thereby decreasing upper airway resistance and reducing OSA severity.^25^, ^26^ Cephalometric assessment of skeletal width, however, requires radiation exposure and does not account for the variable effects of pharyngeal soft tissues (parapharyngeal fat, muscle, and tonsillar tissue) on the transverse width of the pharyngeal lumen.^27^, ^28^

Anthropometric measurements such as body mass index (BMI) and neck circumference (NC) have previously been shown to correlate with the probability of CCC.^29^, ^30^, ^31^, ^32^, ^33^ A preliminary study by Weiner and Kent suggested that measurement of supine pharyngeal width (SPW) in awake patients using a hand‐held caliper was associated with the presence of CCC during DISE as well as HNS response.^33^ Direct measurement of SPW does not require radiation and instead considers the combined contributions of both the skeletal width and pharyngeal soft tissues on transverse airway caliber.

The present study was conducted to prospectively study the anthropometric variables SPW, NC, and BMI in a large, multicenter population of patients seeking HNS therapy. We hypothesized that greater SPW, smaller NC, and lower BMI would associate with a lower probability of CCC during DISE. These simple measurements have the potential to reduce the number of patients needing DISE to ascertain HNS candidacy.

## Methods

### Participants

This study was approved by the IRB at each institution. For sites that did not use local IRBs, all used WIRB‐Copernicus Group (WCG® IRB) (IRB# IRB00000533). This was a prospective, multicenter cohort study (clinicaltrials.gov #NCT05428839) of patients with moderate‐to‐severe OSA and CPAP intolerance scheduled for DISE evaluation for airway surgery after meeting standard clinical anthropometric and polysomnographic indications for HNS therapy. An IRB‐approved waiver of consent and patient information sheet was used at 9 centers, 1 center obtained documented informed consent. OSA severity was assessed by in‐laboratory polysomnography (PSG) or a home sleep apnea test (HST). Demographic, anthropometric, and polysomnographic data were collected for each participant.

### Pharyngeal Width

**Figure 1:**
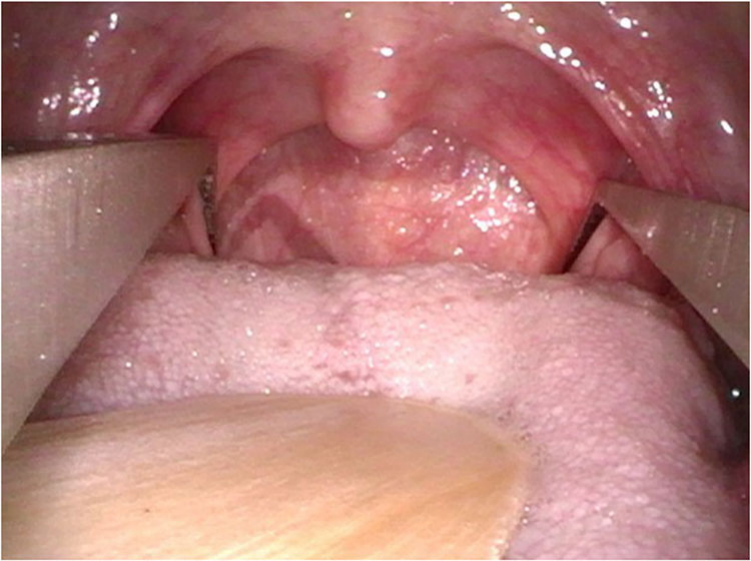
SPW measurement. By permission, Sage Journals from Weiner JS, et al. Supine pharyngeal width is associated with complete concentric palatal collapse during drug‐induced sleep endoscopy and hypoglossal nerve stimulator outcomes. Ear, Nose & Throat Journal. 2022;0(0). SPW, supine pharyngeal width.

Participating surgeons were required to complete remote training in pharyngeal width measurement prior to study initiation. Pharyngeal width was measured prior to DISE using a Castroviejo caliper (Supplemental Figure S1, available online) in the supine position, as previously described (Figure 1).^33^ Briefly, each measurement was taken at the medial border of the palatopharyngeus insertion into the lateral pharyngeal wall (Figure 1). To avoid gagging, surgeons were advised to instruct the patient to breathe with their mouth. Gentle pressure on the dorsal tongue with a tongue depressor is usually applied. The pharynx is observed until the pharyngeal muscles relax. The tips of the caliper do not need to touch the mucosa which reduces the likelihood of gagging. The surgeon was unable to obtain a measurement in 2/600 patients.

### Drug‐Induced Sleep Endoscopy

A standard clinical DISE exam was performed under continuously titrated propofol sedation and scored by the local study surgeon using the VOTE classification.^34^, ^35^, ^36^ Two blinded independent reviewers subsequently scored each recorded examination.

### Data Analysis

#### Variable Definitions

SPW was the main independent variable. Secondary independent variables included demographic and polysomnographic factors known to influence HNS outcomes including age, gender, BMI, and NC.^37^, ^38^ The dependent variable was the presence of CCC during DISE as determined by two blinded, independent reviewers. If there was disagreement regarding presence of CCC between the two independent reviewers, the classification of the study site surgeon was used to establish a single majority classification.

#### Statistical Analysis

Statistical analyses were designed to test the primary hypothesis that greater pharyngeal width is associated with a lower probability of CCC during DISE. The primary analysis used univariate and multiple logistic regression to evaluate associations between pharyngeal width and the presence of CCC after controlling for covariates of interest.

Secondary analyses were designed to evaluate differences in other independent variables between clinical cohorts (CCC and non‐CCC). Student's *t*‐test was used to compare normally distributed continuous baseline characteristic measurements between clinical cohorts and Wilcoxon Rank‐Sum test was used to compare nonnormally distributed continuous baseline characteristic measurements, including pharyngeal width. The Fisher's Exact Test was used to evaluate categorical variables.

Additional analyses were performed using receiver operating characteristic (ROC) curve and area under the curve (AUC) to investigate the sensitivity and specificity of various pharyngeal width measurements for association with CCC. The ROC curve was then used to determine sensitivity, specificity, positive and negative predictive value for the optimal pharyngeal width measurement cutpoint. Similar methods were used to evaluate the optimal BMI and NC cutpoints.

Classification and Regression Tree (CART) analysis was performed, examining the use of the significant and clinically relevant independent variables in combinations to determine if a predictive algorithm could be identified to best predict CCC.

A Weighted Generalized Score Statistic was used to evaluate whether the NPV of a CART model including BMI and NC was significantly different from the NPV if all patients were assumed to have non‐CCC.

Statistical analyses were completed using R Statistical Software (Version 4.3.1; R Core Team 2023). ROC and optimal cutpoint analyses were performed using the cutpointr R package (Version 1.1.2). CART analyses were performed using the rpart R package (Version 4.1.23).^39^ Statistical significance was inferred throughout at a *P* < .05.

## Results

A total of 600 patients across 10 investigational sites were enrolled between May 2022 and January 2024 with 587 completing the study. See Supplemental Table S1, available online for reasons for incomplete data.

CCC was present in 9.9% of patients based on consensus scoring. Consensus of the PI and both reviewers occurred in 79%. At least 1 reviewer agreed with the site investigator VOTE score in 93% of patients. Of the 489 patients classified as non‐CCC per consensus, 13.5% (66) had 1 reviewer that disagreed with the non‐CCC classification and scored the patient as CCC. In general, patients with CCC had a higher BMI, NC, and baseline AHI, and were more likely to be younger and male compared to the non‐CCC patients (Table 1). There were no significant differences in CCC prevalence between study sites.

**Table 1:** Demographics and Baseline Characteristics by CCC Versus Non‐CCC Group

Variable	CCC	Non‐CCC	*P*‐value
N	54	489	
Baseline characteristics			
Age (y)	57.07 ± 11.52 (57.5), 30‐86, N = 54	61.16 ± 11.91 (62), 19‐88, N = 489	.012
BMI (kg/m^2^)*	31.22 ± 3.19 (31.62), 23.47‐36.66, N = 54	28.96 ± 3.42 (29.09), 18.37‐39.98, N = 489	<.001
Neck circumference (cm)	45.47 ± 4.15 (46), 36.83‐55.88, N = 19	40.64 ± 4.7 (40.64), 31.75‐55.88, N = 203	<.001
Baseline AHI (events/h)	39.81 ± 18.94 (34.9), 15‐87.9, N = 54	33.56 ± 14.34 (31), 15‐91.3, N = 489	.034
Gender			
Male	87% (47)	63.6% (311)	<.001
Female	13% (7)	36.4% (178)	
Ethnicity			
Hispanic or Latino	3.7% (2)	3.9% (19)	1
Not Hispanic or Latino	96.3% (52)	96.1% (468)	
Race			
White**	83.3% (45)	89% (435)	.259
Black	11.1% (6)	3.1% (15)	
Asian	0% (0)	1.6% (8)	
American Indian or Alaska Native	0% (0)	0.2% (1)	
Not Specified/Unknown	5.6% (3)	5.7% (28)	
Other race***	0% (0)	0.4% (2)	

Format for numeric variables: Mean ± SD (median), range. Neck circumference measurements collected in inches were converted to centimeters. *P*‐value compares CCC versus non‐CCC groups and was derived using Wilcoxon Rank‐Sum test for numeric variables (due to nonnormality of data) and Fisher's Exact Test for categorical variables, unless noted otherwise.

* *P*‐value derived using Student's *t*‐test.

** *P*‐value compares White versus Non‐White patients. Sample sizes for Non‐White races were too small to statistically compare separately.

*** Other races specified: Hispanic, Puerto Rican.

**Figure 2:**
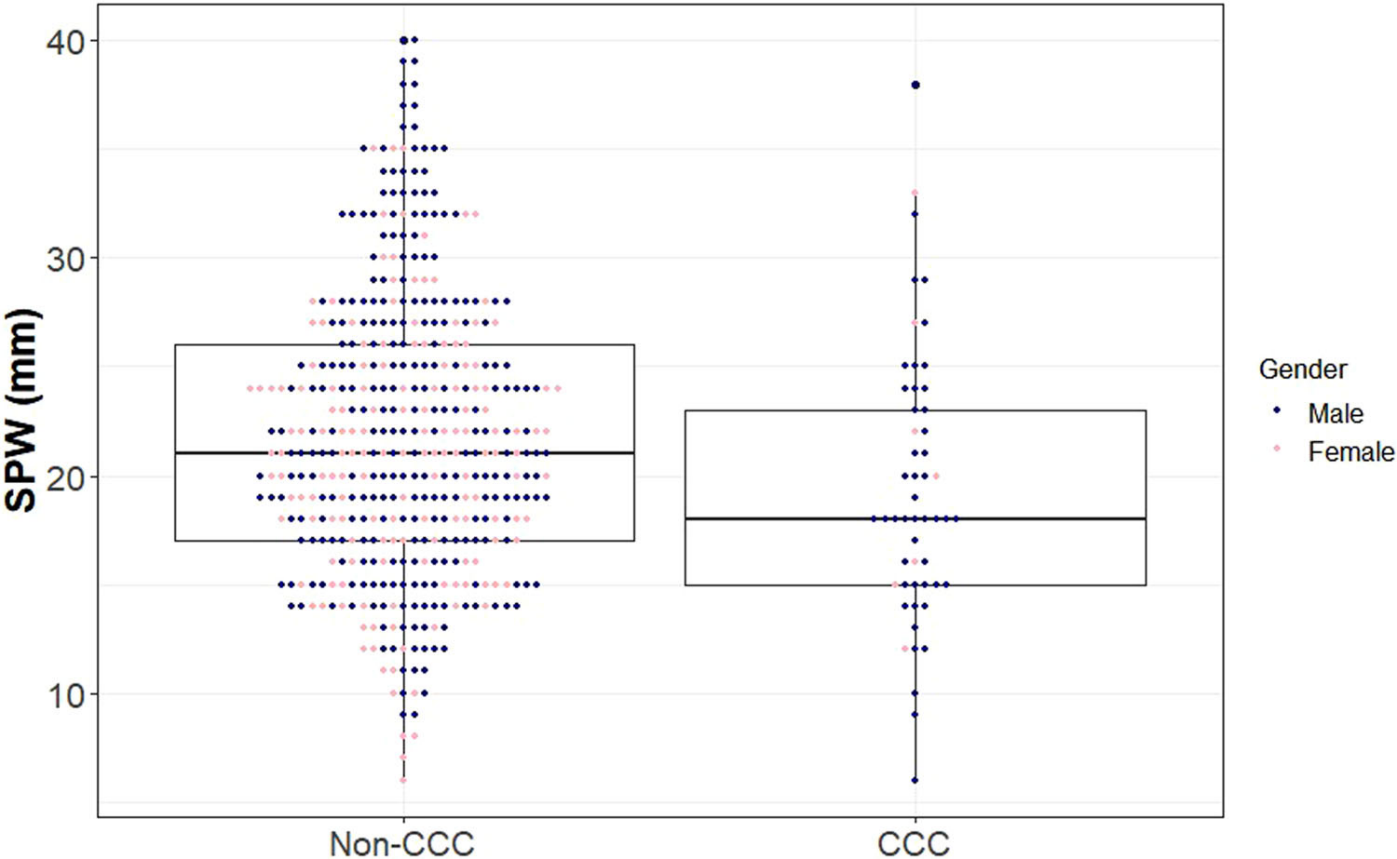
Boxplots of SPW and CCC status. CCC, complete concentric collapse; SPW, supine pharyngeal width.

BMI varied from 18.4 to 40.0 with a mean of 29.2 ± 3.47 kg/m^2^. Patients with CCC had a higher BMI (31.2 ± 3.2 vs 29.0 ± 3.4, *P* < .001) than patients without CCC. For BMI, the selected cutoff was 31.6 kg/m^2^, with a sensitivity of 0.52 (95% CI: 0.38‐0.66), specificity of 0.78 (95% CI: 0.74‐0.82) and NPV of 0.94 (95% CI: 0.91‐0.96) with an AUC of 0.68. NC was collected on 237 of the 587 patients (40%) that completed the study at 5 sites where NC was measured as part of standard clinical care. NC ranged from 31.75 to 55.88 cm (12.5‐22 in). Patients with CCC had a larger NC (45.47 ± 4.15 vs 40.64 ± 4.7 cm or 17.9 ± 1.6 vs 16 ± 1.9 in, *P* < .001) than patients without CCC. For NC, the selected cutoff was 45 cm (17.7 in). This had a sensitivity of 0.63 (95% CI: 0.38‐0.84), specificity of 0.83 (95% CI: 0.77‐0.88), and NPV of 0.96 (95% CI: 0.92‐0.98) with an AUC of 0.78 (Table 2). SPW ranged from 6 to 40 mm with a mean of 21.5 ± 6.5 mm. Patients with CCC had lower SPW (19.4 ± 6.3 mm vs 21.8 ± 6.5 mm; *P* = .008) than patients without CCC (Figure 2). The selected cutoff point for predicting CCC using SPW was 18 mm, with a sensitivity of 0.55 (95% CI: 0.4‐0.68,) specificity of 0.67 (95% CI: 0.63‐0.72) and NPV of 0.93 (95% CI: 0.89‐0.95) with an AUC of 0.61.

**Table 2:** Receiver Operating Characteristic Curve Analysis for BMI, SPW, and NC

Statistic	SPW	BMI	Neck circumference
CCC:Non‐CCC	53:441	54:489	19:203
Optimal cutpoint	18 mm	31.62 kg/m^2^	45 cm
AUC	0.61	0.68	0.78
Sensitivity	0.55 (95% CI: 0.4‐0.68)	0.52 (95% CI: 0.38‐0.66)	0.63 (95% CI: 0.38‐0.84)
Specificity	0.67 (95% CI: 0.63‐0.72)	0.78 (95% CI: 0.74‐0.82)	0.83 (95% CI: 0.77‐0.88)
Positive predictive value	0.17 (95% CI: 0.12‐0.23)	0.21 (95% CI: 0.14‐0.28)	0.26 (95% CI: 0.14‐0.4)
Negative predictive value	0.93 (95% CI: 0.89‐0.95)	0.94 (95% CI: 0.91‐0.96)	0.96 (95% CI: 0.92‐0.98)
Accuracy (correct classifications)	65.99%	75.32%	81.08%

CCC/Non‐CCC status per consensus score determination. Screen failure patients excluded.

Abbreviations: BMI, body mass index; CI, confidence interval; SPW, supine pharyngeal width.

Univariate regression was performed for CCC status by baseline characteristics and SPW. SPW, BMI, age, baseline AHI, gender, and NC were significantly associated with CCC status (Table 3). Multiple logistic regression using stepwise selection per AIC was performed to obtain the most parsimonious model for predicting CCC status, with SPW, BMI, age, baseline AHI, and gender included as independent variables in the full model. SPW, BMI, baseline AHI, and gender were retained in the reduced model (Table 4).

**Table 3:** Univariate Logistic Regression Analysis for CCC Status by Baseline Characteristics and SPW

Variable	Estimate	Std. Error	OR (95% CI)	***z*** value	*P*‐value	N
Supine PW (mm)	−0.06	0.02	0.94 (0.89, 0.98)	−2.54	1.1e−02	494
BMI (kg/m^2^)	0.21	0.05	1.23 (1.13, 1.36)	4.43	9.4e−06	543
Age (years)	−0.03	0.01	0.97 (0.95, 1)	−2.37	1.8e−02	543
Baseline AHI^a^	0.03	0.01	1.03 (1.01, 1.04)	2.87	4.2e−03	543
Gender—Male	1.35	0.42	3.84 (1.81, 9.48)	3.24	1.2e−03	543
Race—White	−0.48	0.39	0.62 (0.3, 1.42)	−1.21	2.2e−01	543
Neck circumference (cm)	0.22	0.06	1.24 (1.12, 1.4)	3.83	1.3e−04	222

Each line in the table represents results from one univariate regression model (intercept estimates not included).

Abbreviations: BMI, body mass index; CCC, complete concentric collapse; CI, confidence interval; OR, odds ratio; SPW, supine pharyngeal width.

^a^ Baseline AHI may have been collected using a Home Sleep Test or in‐lab Polysomnography performed within 24 months of enrollment.

**Table 4:** Multiple Logistic Regression Results for CCC Status (Consensus Score) by Baseline Characteristics and SPW for the Full Cohort and the Subset of Patients With NC Collected. The Most Parsimonious Reduced Models, as Determined by Stepwise Selection Using AIC Criteria, Are Displayed

	Estimate	Std. Error	OR (95% CI)	*z* value	*P*‐value	N
Reduced model for full cohort						
(Intercept)	−8.26	1.71	0 (0, 0.01)	−4.82	1.4e−06	494
Supine PW (mm)	−0.06	0.03	0.95 (0.9, 0.99)	−2.15	3.2e–02	
BMI (kg/m^2^)	0.19	0.05	1.21 (1.1, 1.34)	3.82	1.3e−04	
Baseline AHI (events/h)	0.01	0.01	1.01 (1, 1.03)	1.52	1.3e−01	
Gender—Male	1.27	0.43	3.57 (1.64, 8.97)	2.97	2.9e−03	
Reduced Model for Subset with NC Collected						
(Intercept)	−9.47	2.72	0 (0, 0.01)	−3.47	1.0e−03	193
Age (y)	−0.05	0.02	0.95 (0.91, 0.99)	−2.34	1.9e−02	
Baseline AHI (events/h)	0.03	0.02	1.03 (0.99, 1.06)	1.59	1.1e−01	
Neck circumference (cm)	0.21	0.06	1.24 (1.1, 1.41)	3.35	1.0e−03	

Abbreviations: CCC, complete concentric collapse; CI, confidence interval; OR, odds ratio; SPW, supine pharyngeal width.

Multiple logistic regression to predict CCC status, including SPW, BMI, age, baseline AHI, and gender with the addition of NC in the full model, was also performed. In this regression analysis, age, baseline AHI, and NC were retained in the reduced model after stepwise selection (Table 4). This represented a smaller set of patients (N = 193) due to the limited collection of NC measurements.

**Figure 3:**
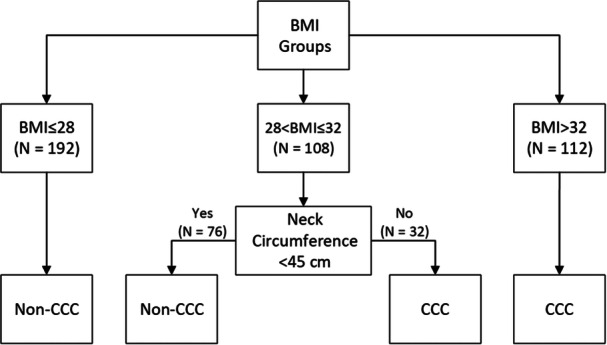
CART analysis combining BMI and NC. BMI, body mass index; CART, Classification and Regression Tree; NC, neck circumference.

The CART model (Figure 3) combining BMI and NC was the most accurate for correct prediction of non‐CCC, correctly categorizing 68.9% of patients overall, with a negative predictive value of 0.96 (Table 5). Adding SPW did not improve the model's performance.

**Table 5:** CART Analysis (Combining BMI and Neck Circumference Thresholds, BMI Groups: </=28, 28‐32, >32)

Statistic	Estimate
Sensitivity	0.71
Specificity	0.69
Positive predictive value	0.19
Negative predictive value	0.96
Accuracy (correct classifications)	68.93%
% Excluded from DISE	65.05%
Error rate	
CCC patients being implanted	4.1%
Non‐CCC patients going to DISE	81.25%

Abbreviations: BMI, body mass index; CART, Classification and Regression Tree; CCC, complete concentric collapse; DISE, drug‐induced sleep endoscopy.

Due to the low rate of CCC in the study, the NPV in which all patients were assumed to be non‐CCC was derived (0.91) and statistically compared to the NPV for the CART model combining BMI and NC (0.96). The NPVs were significantly different (Weighted Generalized Score Statistic = 12.13; *P* = .0005), with an estimated difference of −0.05.

Of note, 1 site (JSW) was observed to have a significant difference in SPW for CCC and non‐CCC patients (14.3 vs 23.8 mm, *P* = .028, N = 58). At that site, the selected cutoff point for predicting CCC using SPW was 13 mm. The sensitivity was 0.75 (95% CI: 0.19‐0.99), specificity 0.94 (95% CI: 0.85‐0.99), NPV 0.98 (95% CI: 0.9‐1), AUC 0.83, and an overall accuracy of 93.1% (Table 6).

**Table 6:** Receiver Operating Characteristic Curve Analyses for BMI, SPW, and Neck Circumference (JSW only)

Statistic	SPW	BMI	Neck circumference
N CCC: N Non‐CCC	4:54	4:55	4:48
Optimal Cutpoint	13 mm	30.03 kg/m^2^	40.64 cm
AUC	0.83	0.76	0.82
Sensitivity	0.75 (0.19‐0.99)	0.75 (0.19‐0.99)	1 (0.4‐1)
Specificity	0.94 (0.85‐0.99)	0.73 (0.59‐0.84)	0.58 (0.43‐0.72)
Positive predictive value (PPV)	0.5 (0.12‐0.88)	0.17 (0.04‐0.41)	0.17 (0.05‐0.37)
Negative predictive value (NPV)	0.98 (0.9‐1)	0.98 (0.87‐1)	1 (0.88‐1)
Accuracy (correct classifications)	93.1%	72.88%	61.54%

Abbreviations: BMI, body mass index; SPW, supine pharyngeal width.

## Discussion

This study demonstrated that NC, BMI, and SPW independently associated with complete CCC. When analyzed together, a classification and regression tree model including BMI and NC correctly categorized patient CCC status over two‐thirds of the time, yielding a negative predictive value of 0.96. The Weighted Generalized Score Statistic suggests that this is statistically different than the NPV of 0.91 if it were assumed that all patients would not have CCC, given the low incidence of CCC in this study. Our results suggest that these inexpensive and easily obtained clinical measurements may hold significant value for predicting CCC status without the time and expense of a drug‐induced sleep endoscopy.

Currently, all patients meeting eligibility requirements for HNS placement must undergo DISE to exclude the presence of CCC based on patient selection criteria from the pivotal Stimulation Therapy for Apnea Reduction (STAR) trial.^9^ The STAR criteria were derived from small early‐phase studies showing decreased HNS therapy response in patients with CCC.^16^, ^35^ This adds an extra step in the evaluation of every candidate. In the United States, DISE costs range from $2861 and $7942.^18^ Bypassing this step in most patients would reduce costs per patient implanted.

In contrast, BMI and NC are easily obtained and already utilized in many practices. Measuring SPW takes about 1 minute in experienced hands. Measurements of maxillary width do not account for soft tissue volumes and would thus only provide partial insight into site and mechanism of airway collapse while SPW would theoretically be influenced by both maxillary width as well as total soft tissue volume. It should be noted that soft tissue volume includes both muscle and fat. Previous investigators have shown that the airway is smallest in the retropalatal oropharynx and that the size of the airway at this level is best predicted by the thickness of the lateral pharyngeal wall musculature with a lesser contribution from parapharyngeal fat volume. They also demonstrated that in OSA patients, the shape of the airway at this level was oval with flattening in the lateral dimension rather than an ovoid shape narrowest in the AP dimension.^28^ This would provide theoretical support for the use of SPW since it is a measurement of airway width at this narrowest point.

**Figure 4:**
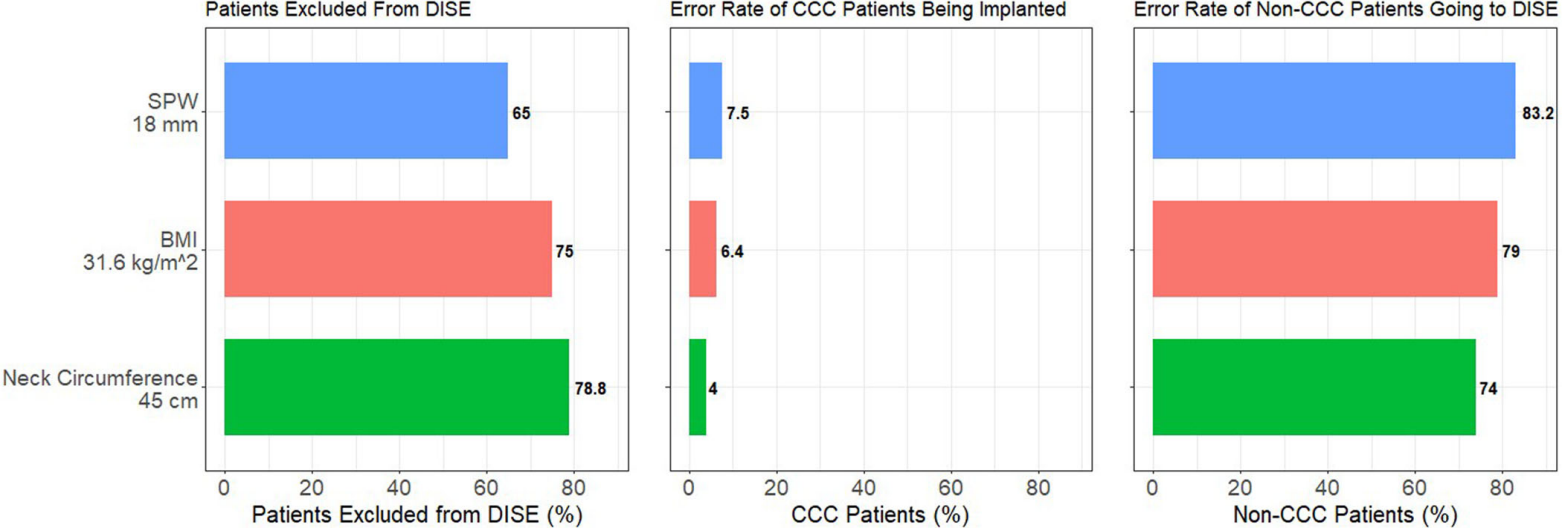
Screening implications using SPW, BMI, and NC cutpoints. BMI, body mass index; DISE, drug‐induced sleep endoscopy; NC, neck circumference; SPW, supine pharyngeal width.

In the present study, using the chosen cutpoints for SPW, BMI, and NC, 65%, 75%, and 79% of patients, respectively, could potentially forgo DISE and proceed to HNS. Of those patients bypassing DISE, 7.5%, 6.4%, and 4%, respectively, would be falsely assumed to not have CCC and would potentially go on to HNS implantation (Figure 4). Other studies have also investigated the possibility of predicting CCC using various methods. Schoustra and colleagues evaluated the association of BMI and CCC in 1761 patients. The CCC group had a higher BMI as well as NC. The optimal BMI cutpoint was 29.4 kg/m^2^ for males, and 29.5 kg/m^2^ for females with a specificity of 78% and 75%, respectively and AUC of 0.65. In that study, NC did also associate with CCC. Using a NC in males of 16.4 in. and 14.1 in. in females, there was a NPV of 86.3% and 90.6%, respectively and AUC of 0.62 in males and 0.53 in females. However, in their CART analysis including height, tonsil status, NC, and BMI the AUC increased to 0.82.^32^ In that study, the cutpoints for BMI and NC were lower than in the present study.

Similarly, Gao et al investigated the association of BMI and NC with CCC in a series of 289 patients. Using a cutpoint of 34.8 kg/m^2^ for males, NPV was 90.6% and for females, using a cutpoint of 36.4 kg/m^2^, 98.4% with an overall AUC of 0.7. It should be noted that these cutpoints, are considerably higher than in the present study or in the Schoustra study discussed above. NC was also associated with CCC for both males and females. The optimal NC cutpoint was 18 in. in males and 15 in. in females with a NPV of 88.3% and 100% with an AUC of 0.62 and 0.72, respectively.^30^

Taken together with the results of the present study, it appears that both BMI and NC do associate with CCC. In this study, the NPV of 94% for BMI and 96% for NC in the present study are higher than the other referenced studies.

The reported cutpoints in this study would misclassify 4% to 7% of patients with true CCC as non‐CCC. This compares to a 13.5% disagreement rate in DISE scoring such that using DISE, 13.5% of patients would go on to HNS per 2 reviewers even though 1 reviewer would classify the patient as CCC. This indicates subjectivity of CCC interpretation even amongst physicians experienced in performing DISE.

In this study, 90.1% had nonconcentric palatal collapse. Thus, any screening tool to replace DISE would have to have a NPV substantially greater to be clinically relevant. A NPV of 0.96 is statistically and clinically greater and could be high enough to determine HNS candidacy without DISE.

Data from the most experienced investigator's site showed a much greater difference in SPW between patients with CCC and those without (14 vs 24 mm) as well as greater specificity, sensitivity, NPV, and AUC than the total data set. Using SPW, 93% of patients were correctly classified as CCC versus non‐CCC. At that site, a NPV of 98% for CCC could be high enough to allow bypassing DISE for most patients unless performance of DISE was desired for additional information. While the reason for this difference is not known, it could be due to greater experience taking the SPW measurement. It is possible that some of the measurements by other study authors were taken before the pharyngeal muscles relaxed. With added training and experience, the other investigators might achieve increased accuracy, making SPW a reasonable means of excluding patients with CCC.

Several limitations of the present study should be acknowledged. First, the lack of sufficient numbers of females with CCC prevented statistical analysis of gender differences. Second, study investigators received training on the collection of SPW from printed and video sources but not in‐person instruction. This may have introduced inconsistency in the measurement of SPW which would have reduced effect sizes. Finally, the present study does not include outcomes with HNS. However, a sub‐study is in progress on patients from this study who went on to HNS implantation. This will help determine if the study variables from the present study associate with HNS outcomes. If so, this would make their utility greater than simply as a means of predicting CCC.

There are also noteworthy strengths of this study. As a prospective study, observer bias should be minimized. There is also a relatively large sample size from a geographically and racially diverse population. Finally, there was good agreement between the site investigators and the independent reviewers for the presence of CCC which speaks to the experience of the study investigators in performing DISE.

## Conclusion

BMI, NC, and SPW all associate with CCC and could potentially be used to determine HNS candidacy instead of DISE for most patients. Although a small percentage of patients would go onto HNS who would have had CCC had DISE been performed, the considerable cost savings as well as the elimination of the added burden of an endoscopy with sedation, the interpretation of which having some subjectivity, could make this a reasonable tradeoff.

## Author Contributions

**Jordan S. Weiner**, **MD**, concept and study design, acquisition, analysis and interpretation of data, drafting and revision of the manuscript, final approval of the manuscript; **Eugene G. Chio**, **MD**, acquisition and interpretation of data, revision of the manuscript, final approval of the manuscript; **Raj C. Dedhia**, **MD**, **MSCR**, acquisition and interpretation of data, revision of the manuscript, final approval of the manuscript; **Colin T. Huntley**, **MD**, acquisition and interpretation of data, revision of the manuscript, final approval of the manuscript; **Michael J. Hutz**, **MD**, acquisition and interpretation of data, revision of the manuscript, final approval of the manuscript; **Phillip A. Huyett**, **MD**, acquisition and interpretation of data, revision of the manuscript, final approval of the manuscript; **Daniel J. Lee**, **MD**, acquisition and interpretation of data, revision of the manuscript, final approval of the manuscript; **Phillip LoSavio**, **MD**, acquisition and interpretation of data, revision of the manuscript, final approval of the manuscript; **Ilya Perepelitsyn**, **MD**, acquisition and interpretation of data, revision of the manuscript, final approval of the manuscript; **Ryan J. Soose**, **MD**, acquisition and interpretation of data, revision of the manuscript, final approval of the manuscript; **David T. Kent**, **MD**, study design, acquisition, analysis, and interpretation of data, drafting and revision of the manuscript, final approval of the manuscript.

## Disclosures

Preliminary results of this manuscript were presented at the International Surgical Sleep Society meeting on September 29, 2023 in Nashville, TN.

### Competing interests

Jordan S. Weiner, MD: Consultant, speaker for Inspire Medical Systems, Inc. Eugene G. Chio, MD: Consultant for Inspire Medical Systems and Nyxoah SP. Raj C. Dedhia, MD: Research grants: Inspire Medical Systems, Nyxoah Medical, Cryosa Medical; Consultant for Inspire Medical Systems, Nyxoah Medical. Colin T. Huntley, MD: Research support Nyxoah SP and Inspire Medical Systems. Consultant Nyxoah SP, Inspire Medical Systems, and Lunair. Michael J. Hutz, MD: Consultant, Physician Medical Advisory Board for Inspire Medical Systems, Inc., Consultant, Nyxoah Medical LLC, Consultant, Avivomed, Consultant, Lunair Medical LLC. Phillip A. Huyett, MD: Research support (Nyxoah, Inc.); Research support and educational consultant (Inspire Medical Systems). Daniel J. Lee, MD: No conflict of interest. Phillip LoSavio, MD: Consulting, Inspire Medical Systems. Ilya Perepelitsyn, MD: No conflict of interest. Ryan J. Soose, MD: Consultant/scientific advisory for Cryosa Inc., Inspire Medical Systems, XII Medical; Institutional research support from Inspire Medical Systems. David T. Kent, MD: Consultant Restera, Scientific Advisory Boards Nyxoah SA, Intellectual Property Interests: listed as an inventor on US and international patents and applications owned by Vanderbilt University and licensed to Nyxoah SA, Research Support, Inspire Medical Systems, Inc., Restera, Nyxoah SA.

### Funding source

Inspire Medical Systems, Inc., Golden Valley, MN.

## Supporting information


**Supplemental Figure** 1**:** Castroviejo caliper*. * By permission, Sage Journals from Weiner JS, et al. Supine Pharyngeal Width Is Associated With Complete Concentric Palatal Collapse During Drug‐Induced Sleep Endoscopy and Hypoglossal Nerve Stimulator Outcomes. Ear, Nose & Throat Journal. 2022;0(0).

**Supplemental Table** 1**:** Screen Failures.
