# Supplementary figures and images for: Anthropometric Measurements Inform Complete Concentric Collapse Status in Patients With Obstructive Sleep Apnea
Source: OTO Open. 2026 May 5;10(2):e70245. doi: 10.1002/oto2.70245 (PMC13141678; doi:10.1002/oto2.70245)

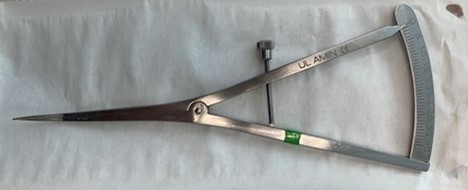

Supplement: Supplementary file 1 — Supplemental Figure 1: Castroviejo caliper*. * By permission, Sage Journals from Weiner JS, et al. Supine Pharyngeal Width Is Associated With Complete Concentric Palatal Collapse During Drug‐Induced Sleep Endoscopy and Hypoglossal Nerve Stimulator Outcomes. Ear, Nose & Throat Journal. 2022;0(0). [file OTO2-10-e70245-s001.jpg]
