# Supplementary material for: Anthropometric Measurements Inform Complete Concentric Collapse Status in Patients With Obstructive Sleep Apnea
Source: OTO Open. 2026 May 5;10(2):e70245. doi: 10.1002/oto2.70245 (PMC13141678; doi:10.1002/oto2.70245)
Supplement: Supplementary file 2 — Supplemental Table 1: Screen Failures. [file OTO2-10-e70245-s002.docx]

| **Reason** | **Number of patients** |
| --- | --- |
| Did not meet eligibility criteria | 7 |
| Investigator unable to complete SPW measurement | 2 |
| Subject deemed “unfit” to participate | 1 |
| No SPW available | 2 |
| VOTE score not completed | 1 |
